# Experimental design for single-cell RNA sequencing

**DOI:** 10.1093/bfgp/elx035

**Published:** 2017-11-08

**Authors:** Jeanette Baran-Gale, Tamir Chandra, Kristina Kirschner

**Affiliations:** 1 The Institute of Genetics and Molecular Medicine, University of Edinburgh, Western General Hospital, Crewe Road, Edinburgh, UK; 2 Institute for Cancer Sciences, University of Glasgow, Switchback Road, Glasgow, UK

**Keywords:** single-cell RNA sequencing, Smart-Seq 2, 10x Chromium, Drop-Seq, experimental design

## Abstract

Single-cell RNA sequencing (scRNA-seq) has opened new avenues for the characterization of heterogeneity in a large variety of cellular systems. As this is a relatively new technique, the field is fast evolving. Here, we discuss general considerations in experimental design and the two most popular approaches, plate-based Smart-Seq2 and microdroplet-based scRNA-seq at the example of 10x Chromium. We discuss advantages and disadvantages of both methods and point out major factors to consider in designing successful experiments.

## Introduction

Single-cell genomic technologies have revolutionized the way scientists can interrogate heterogeneous tissues or rare subpopulations of cells. Single-cell RNA sequencing (scRNA-seq) has been at the forefront of method development both in the laboratory and computationally to provide robust methods for downstream data analysis.

A recent flurry of papers highlighted the potential of this technology. In haematology, single-cell transcriptomics was applied to elucidate age-related changes to the blood system and address heterogeneity of ageing [1, 2]. Signalling pathways critical to the development of cerebral cortex were recently uncovered by low coverage single-cell mRNA sequencing [3], and liver tissue was spatially reconstructed based on transcriptomes of single hepatocytes, highlighting division of labour within different spatial zones of the organ [4]. While scRNA-seq offers new avenues to explore thus far unanswerable questions, it is important to consider experimental design carefully before conducting the study to avoid confounding factors and to be able to draw sound conclusions based on biological variation rather than technical artefacts associated with batch processing of samples.

In this review, we discuss the experimental design for single-cell transcriptome analysis based on the two most popular protocols used for scRNA-seq: Smart-seq2 and 10X Chromium 3′ sequencing. We highlight pros and cons of each method and summarize important considerations to help design successful single-cell transcriptome experiments.

## Smart-Seq 2

One of the most successful methods for single-cell gene expression was reported by Picelli and colleagues [5]. They optimized the SMART (Switching Mechanism at 5′ End of RNA Template) workflow, which is based on high-fidelity reverse transcription, template switching and preamplification for increased complementary DNA (cDNA) yield for single-cell analysis. This protocol takes 2 days hands-on wet-lab work where clean-up steps can be automated if a robot is available. Typically, this protocol provides good coverage of the transcriptome with rarer transcripts being detectable and does not need any specialist equipment. Therefore, it is readily available to a wide community of researchers. However, because of the manual nature of the protocol, processing of cell numbers is limited to the hundreds with either one 96 or one 384 well plates being processed at the same time, causing technical variability from experiment to experiment because of the many pipetting steps. Therefore, batch effects need to be considered in the experimental design. As with other methods relying on oligo dT priming, Smart-Seq2 transcriptomes show a significant 3′-prime bias.

Cell culture-based approaches allow for easy separation of single cells using routine trypsinization methods, with liver perfusions leading to single-cell solutions and blood readily providing access to single cells. Before conducting a single-cell experiment, it is recommended to trial single-cell separation methods and assess cell viability, for example processing a polymerase chain reaction (PCR) strip of eight cells, instead of a full 96 well plate. Cell separation for Smart-Seq2 is typically achieved by fluorescence-activated cell sorting (FACS), enabling a wide variety of cell types to be isolated in the same experiment if necessary. Single cells are dropped into 96 or 384 well plates containing a hypotonic lysis buffer containing Triton-X100 with excess ribonuclease inhibitor to stabilize RNA [5]. Cell isolation should, therefore, be performed as fast as possible with all downstream work being carried out on ice. However, depending on cell type, stronger lysis buffers might need to be used and trialled before completing a full set of experiments to ensure compatibility with the downstream protocol. Besides, we observed cell-type-specific differences in recovery after FACS sorting, possibly because of cell size, with larger cells resulting in fewer cells recovered. The flow rate and size of nozzle can be adjusted to improve the sort, increasing recovery or/and avoiding doublets. Once sorted, plates can be spun down and stored at −80 °C. In our experience, storage of plates for 6 months and longer yielded good quality cDNA. Depending on the FACS sorter, index sorting might be available and is highly recommended for downstream analysis and QC steps, as the transcriptome of individual cells can be linked to the expression of cell surface protein markers, cell cycle status, cell size or granularity. In addition, empty wells and wells containing more than one cell (=doublets) can be detected by indexing during the sort and wells either refilled or excluded from downstream processing or analysis.

A pre-PCR, amplicon-free environment is essential for successful separation of high-quality cDNA from single cells to avoid contamination and can either be achieved by providing a dedicated pre-PCR room or by using a pre-PCR bench mounted hood together with pipettes and a thermal cycler dedicated to pre-PCR work. Once cDNA is obtained, all follow-on clean-up steps and library work can be performed on a standard laboratory bench. Overall, Smart-Seq 2 is a robust and reliable method for single-cell transcriptome profiling using little or no specialist equipment. As the field continues to evolve, methods will continue to push the envelope increasing the number of cells captured at single-cell resolution in an experiment. One such evolving method involves the use of a combinatorial indexing scheme, in which a series of unique barcodes are sequentially added to pools of cells that are randomly sorted into wells between each barcode addition [6].

## Microfluidics-based approaches

Alternatives to the Smart-Seq 2 workflow are microfluidics methods, which use a similar molecular biology (they also rely on template switching, for example), but are different in their cell capture and throughput. During the droplet-based workflow [7, 8] individual cells are encapsulated into nanoliter droplets containing DNA-barcoded reads for reverse transcription. For cDNA, recovery droplets are broken up, and cDNA is subjected to library preparation. This method allows for thousands of cells to be profiled simultaneously, but it initially required specialist, custom-designed equipment, which made it difficult to access. Recently, several microdroplet-based instruments were released offering convenient platforms for droplet-based single-cell analysis with a tunrover of 2–3 days. For example, BioRad offers a ddSeq single-cell isolator, which is linked to the Illumina Nextera kits. The InDrop by 1CellBio offers another alternative microfluidics system. The Fluidigm C1 platform offers low-throughput microfluidics with the advantage of being able to visually control for empty wells or doublets following the capture. However, the most commonly used platform at the moment is the single-cell controller from 10x Genomics, and we will discuss its properties further in this review. Microdroplet-based approaches are designed to assess large numbers of cells and lend themselves for tissue profiling and detection of new cell types.

Important factors for successful, high-quality data generation using microdroplet technology are the quality of cells and the cell numbers used per experiment. Ideally, only live cells are fed into the system, and therefore, quick isolation and mild dissociation of cell types is essential and should be trialled before committing to the actual experiment (see above). Viability can reliably be tested using dye exclusion methods or FACS sorting using a live/dead cell marker before loading of cells onto the chip. The number of doublets encapsulated in the same droplet increases (∼0.8%/1000 cells) with rising cell numbers and needs to be considered. Recently, Alles and colleagues [9] reported a methanol-based fixation method for single-cell transcriptome profiling using microfluidics. This method ensures preservation of transcriptome properties and is particularly useful when working with rare clinical samples or time course samples, where downstream processing needs to occur at the same time.

## Comparison of Smart-Seq2 and 10x Chromium 3′ sequencing platforms

Deciding on a platform ultimately depends on the question addressed. An outline of features for both platforms is summarized in Table 1. Studying blood is a good example of a tissue where both Smart-Seq2 and 10x Chromium approaches have successfully been used. Blood offers the advantage of access to single-cell solutions without requiring additional tissue dissociation steps making it easy and quick to load a 10x controller, and data sets on several thousand peripheral blood mononuclear cells are available on the 10x Genomics website. However, it is often necessary to enrich for rare subpopulations [1, 2, 10].

**Table 1.** Comparison of Smart-Seq2 and 10x Chromium platform

Company	Protocol	Cost per cell US$	Number of cells	Characteristics	Library	Sequencing
	SmartSeq2	11	96–384	full length capture	Nextera	HiSeq 2500 or 4000
10x Genomics	Chromium	12 for 1000 cells per run	100–100 000	3′-tag method	10x Genomics	HiSeq 2500 or 4000

**Figure 1.**
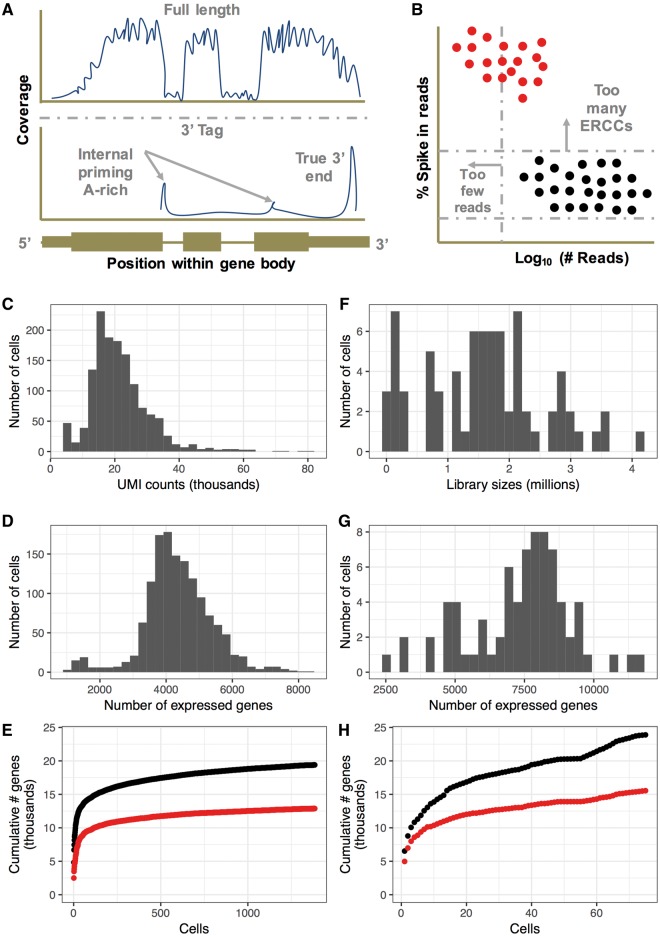
Comparison of 10x and SMART-seq2. **(A)** The protocols differ in the fraction of the gene covered by reads. While full-length protocols (such as Smart-Seq2) have reads covering the entire gene body, 3′-tag methods (such as 10x) concentrate reads upstream of the polyA tail or internally upstream of A-rich regions of the transcript. **(B)** Percentages (%) of reads aligning to ERCCs can be used in Smart-Seq2 data sets to identify high-quality cells. In comparison, non-ERCC data sets rely on library size/total UMI counts and the number of features detected. **(C–E)** Characteristics of a representative 10x data set of 1384 growing IMR90 cells. **(F–H)** Characteristics of a representative Smart-Seq2 data set of 75 growing IMR90 cells. **(E and H)** The cumulative number of genes detected at > 0 reads (black) or > 1 UMI or 10 reads (red) across cells in each data set.

Depending on the method used to generate scRNA-seq data sets, some distinct characteristics of the data become apparent, which might influence experimental design. A key difference between Smart -Seq2 and the 10x Chromium protocol lies in the way the RNA is processed to cDNA. Smart-seq2 captures the full-length mRNA, although with significant 3′ bias because of oligo dT primers used during cDNA generation, while the 10x protocol is based on a 3′-tag sequencing method (Figure 1A). Accordingly, it is important to consider the aim of the study when selecting a method for single-cell RNA-seq. For example, full-length capture is needed for studies concerned with isoforms or gene fusions, while 3′-tag methods can capture more cells and thus give an aggregate view of the transcriptional heterogeneity of a given cell population.

As part of the quality control process, spike-in controls are often used to assess differences in RNA content between individual cells in the same experiment [11, 12]. The usefulness of spike-in controls remains highly debated, with the main criticisms of its use including (1) uncertainty about variation in spike-in concentration between cells, (2) concerns that the synthetic spike-ins will not be processed in a manner similar to endogenous RNAs and (3) difficulty in finding the correct spike in concentration per sample to normalize the data without under or overwhelming the signal with reads associated with spike-ins. A recent paper by Lun *et al.* [13] assessed some of these main concerns and demonstrated that the variance in the spike-in concentration and processing is a small part of the total technical variation. We find spike-in controls useful when determining empty wells or dead cells within a sequencing experiment, as high External RNA controls Consortium (Ercc) content correlates with low-quality data and is usually an exclusion criterion (Figure 1B). However, other methods such as low numbers of transcripts can be used in a similar fashion; hence, the usefulness of inclusion of Ercc spike ins should be determined individually, as sequencing output can be reduced depending on the level of spike-ins. In summary, properly used, Ercc spike-ins can improve RNA normalization, particularly in data sets where total RNA content varies across cells and to detect multiples, which often have unusually high numbers of transcripts. However, optimization is necessary to ensure that the ratio of spike-ins is in the correct range. While both Smart- Seq2 and the 10x Chromium approaches allow using spike-in controls, spike-ins are more commonly used in the Smart-Seq2 protocol.

Finally, the protocols differ in the inclusion of unique molecular identifiers (UMIs) as a means to correct for amplification bias [14]. Protocols for Smart-Seq 2 and other full-length approaches make the inclusion of UMIs difficult, as each ‘full length’ transcript is fragmented following reverse transcription, and each fragment would need to be linked to the single UMI for that transcript. In the 10x system a 10 bp UMI is included in each read at the beginning of the protocol facilitating the calculation of estimated molecule counts and of sequencing saturation through the examination of UMI duplicates. The fraction of UMI duplication in UMI-based data sets depends on the depth of sequencing, with standard rates of duplication for representative data sets exceeding 60%. Thus, the inclusion of UMIs can be particularly useful to eliminate overrepresentation of certain gene loci for absolute quantification. However, Tung and colleagues [15] recently investigated the use of UMIs on the C1 Fluidigm platform to account for batch effects. They conclude that the use of UMIs, although useful at correcting for amplification bias, cannot be used as a completely unbiased estimator of gene expression [15].

Major differences between the two protocols include: (1) the 10x protocol often captures more cells than the Smart-seq2 protocol, and thus library sizes can differ by several orders of magnitude depending on the sequencing design (Figure 1C and F). (2) Smart-seq2 data sets often capture more genes per cell than their 10x counterparts (Figure 1G and D). (3) Despite having fewer cells, Smart-seq2 data sets, in aggregate, are more complex than the 10x data sets, although many of these genes may be below the limit of detection needed to make meaningful observations about expression in that cell (Figure 1E and H). Overall, the 10x Chromium system offers convenience and requires less manual handling compared with Smart-Seq2, simplifying the collection of data sets containing large numbers of cells. However, as one increases the numbers of cells in the data set, one also increases the required depth of sequencing, sacrifice the complexity of the library and lose the ability to easily customize the workflow.

## Experimental design considerations

### Avoiding technical biases

**Figure 2.**
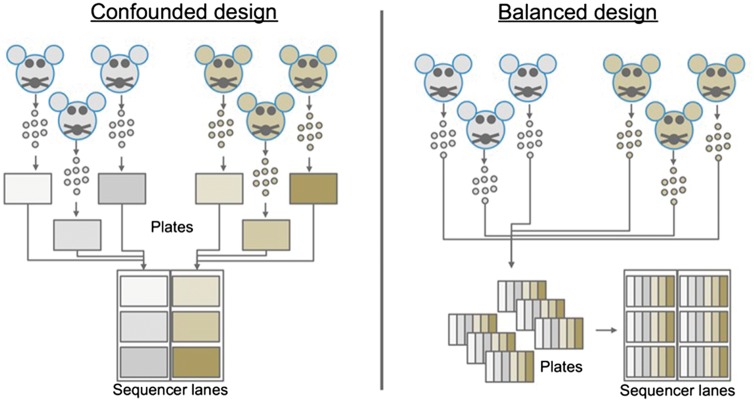
Experimental design examples. In the confounded design, cells are isolated from each sample onto separate plates, processed at potentially different times and the two groups (indicated by different colors) are sequenced on separate lanes of the sequencer. In the balanced design on the right, all samples are evenly distributed across all stages of the experiment, thus reducing the sources of technical variation in the experiment.

Experimental design papers span the evolution of transcriptome-wide methods beginning with microarray studies and continuing to the present with the introduction of single-cell RNA-seq [15–21]. Sound experimental design begins with three principles formalized by R. A. Fisher in 1935: replication, randomization and blocking. Therefore, use of biological replicates, random assignment of groups and a balanced block design are essential factors underlying a successful sequencing experiment (Figure 2).

Often, the most difficult principle to adhere to is the blocking principle. In the context of RNA sequencing, the blocking principle is most frequently violated by a need to separate the experiment into batches, thus introducing a source of technical variation. To avoid confounding factors, batches should be constructed such that the experimental conditions are evenly or at least randomly spread across all samples. In an unblocked design, it is possible to mask biological variation with technical variation resulting in a confounded experiment. For example, imagine an experiment where cells are collected from mice under two experimental conditions (*N* = 3, Figure 2). In a confounded design, cells from a single mouse/condition group would be sorted onto a single plate, and the library for cells on that plate would be prepared in one batch. Finally, the libraries are sequenced in one lane, while the libraries from the other condition are sequenced in another (Figure 2). In this example, it is not possible to tell whether the differences in gene expression are because of the experimental condition or the technical variation introduced by the confounded experimental design. This experiment could be redesigned using a balanced block design by having samples from each of the six mice on each plate and lane of the sequencer. FACS sorting for Smart-seq2 allows to select individual wells to be filled with cells, making a balanced approach easy. For the 10x Chromium, one needs to ensure to process samples on different chips during the initial encapsulation and for further downstream handling to balance the experiment. When the number of conditions exceeds the number of samples in a batch, it is still possible to design a balanced experiment that limits confounding factors [11, 16]. In reality, it is well documented in the literature that failure to design experiments in a balanced manner results in artefacts. Typically, a paper is first published identifying differences in expression between conditions [22–25], followed by the eventual release of a second or series of publications reanalysing the data while properly accounting for the contribution of batch effects, concluding that the study in the original study was confounded [19, 26, 27].

In summary, failing to balance conditions and samples across all stages of the experiment will result in the introduction of additional sources of technical variation associated with batch preparation of libraries or sequencing. Batch effects can be introduced at any stage of the experiment by unbalanced batch processing of samples, libraries or unbalanced distribution of samples across sequencing lanes and are therefore important factors to consider. To deal with batch effects in single-cell data, novel tools are required. Hagverdi and colleagues [28] recently presented a new tool dealing with batch effects based on the detection of mutual nearest neighbours in high-dimensional expression space. Here, neither predefinition of the population nor composition thereof is necessary. Instead, a subpopulation needs to be shared between batches [28].

### Deciding on appropriate cell numbers

**Figure 3.**
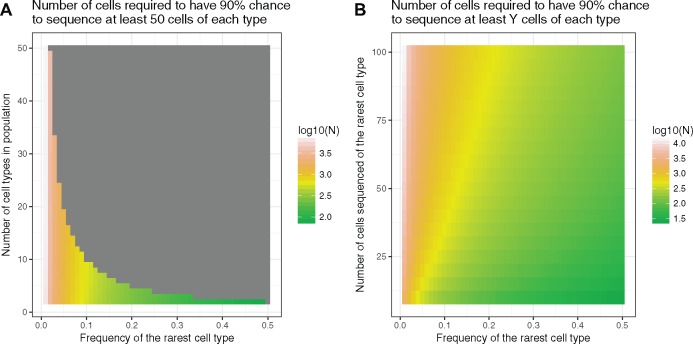
Estimate of cells required for experiments with various parameters. **(A)** The plot shows the log10(#Cells) required to capture at least 50 cell types based on the parameters on the X- and Y-axes. **(B)** The plot shows the log10(#Cells) required to capture the number of cells on the Y-axis if the population consists of 20 cell types.

One consideration during the experimental design process is determining the number of cells that need to be sequenced per experiment. This parameter can be estimated based on the expected heterogeneity of all cells in a sample, the minimum frequency expected of a particular cell type within the sample and the minimum number of cells of each type desired in the resulting data set. With this information, a negative binomial distribution can be used to estimate the number of cells likely to capture at least a set number of cells from your rarest cell type. The number of cells captured from the rarest cell type can be modelled as a negative-binomially distributed random variable. Next, we estimate a lower bound on the probability of capturing at least a certain amount of cells from any of the more abundant cell types in the same experiment by observing that that probability is greater or equal to that of the probability of capturing rarest cell type. Therefore, the probability of sequencing at least *k* cells of each type is greater or equal to the product of those probabilities. For example, if we sequence a mixture of ∼10 cell types where the frequency of the rarest cell type is ∼0.03, then we would need to sequence ∼2200 cells to have a 90% chance of capturing at least 50 of those rare cells (Figure 3A and B).The Satija lab now provides an online tool to estimate cell numbers based on number of cell types and diversity (www.satijalab.org/howmanycells). In cases where no previous knowledge exists about the heterogeneity of the population, the best solution is to perform a pilot-level study with high cell number and lower sequencing depth. In such a study, the 10x platform could be used with an input of 10 000 cells isolated from your population of interest, and the recommended minimum sequencing depth of 50 000 raw reads per cell.

Another design consideration is the sequencing depth of the experiment. Accurate estimation of the sequencing depth per experiment requires foreknowledge of both total mRNA content in individual cells and the diversity of mRNA species in those cells. These parameters are difficult to estimate before conducting the actual experiment. Svensson and colleagues [29] provide a useful guideline in a recently published study, where they performed a comprehensive analysis of single-cell data consisting of 34 unique experiments using 16 different protocols and five species. They found that while 250 000 reads per cell are sufficient for accuracy, 1 million reads per cell were a good target for saturated gene detection [29]. While sequencing depth requirements may vary from experiment to experiment, these figures provide a good estimate to selecting the sequencing depth in most cases. Finally, as statistics continue to be collected regarding single-cell experiments better estimates of required depth and heterogeneity become available. Dr James Hadfield and colleagues have pioneered a Web portal to collect quality control statistics from the community (http://10xqc.com), which currently collects statistics about 10x data sets but will hopefully expand to other single-cell experiments in the future.

## Conclusions

Overall, several factors need to be considered before choosing a method for scRNA-seq. First, costs and availability of equipment are important factors before starting an experiment. Secondly, limitations in cell numbers or profiling of large numbers of cells together with required flexibility in terms of experimental approach are other factors to determine. Finally, coverage for downstream analysis plays into the experimental design process with all confounding factors to be carefully considered before the start of an experiment. In some instances, using both methods to answer the same questions might be the most appropriate way to ensure optimal downstream analysis. For example, a superficial exploratory experiment could be designed using 10x technology to provide estimates of heterogeneity within a cell population to then guide a more in-depth sequencing approach on lower cell numbers to capture more detail in the analysis. New technical developments in the field of single-cell genomics will broaden the spectrum of experimental approaches possible over time. In any case, new insights into cellular and tissue properties on the single-cell level will be an exciting area of investigation over the next few years.

### Data and methods

Two unpublished IMR90 data sets from the Chandra lab were used to provide a comparison between the 10x and Smart-seq2 protocols. 10x: Growing IMR90 cells were processed using the 10x single-cell 3′ protocol (V2; 10x Genomics). In total, 96 cells were loaded and processed according to the standard V2 protocol. The resulting libraries were aligned to the GRCh38 genome, and gene counts were quantified using the CellRanger pipeline (VX). Smart-seq 2: Growing IMR90 cells were sorted into two 96 well plates and subjected to the Smart-seq protocol as described [5]. Data were aligned and to the GRCh38 genome using TopHat, and gene counts were quantified over the same transcriptome as the 10x dataset using SeqMonk (1.38.2; https://www.bioinformatics.babraham.ac.uk/projects/seqmonk/).

**Key Points**

- Smart-Seq 2 is a flexible, low-throughput method without specialist equipment requirements.
- 10x Chromium platform allows for large cell numbers to be assessed simultaneously with specialist equipment required.
- A balanced experimental design is essential to avoid technical artefacts and analyse biological signatures in subsets of cells.

## Notes

**Jeanette Baran-Gale** received her PhD from the University of North Carolina at Chapel Hill characterizing the dynamics of mRNA and miRNA responding to estrogen stimulation in breast cancer. Her research focuses on understanding complex aspects of gene expression regulation. Her current projects investigate mRNA expression using the 10x Chromium platform in thymic epithelial cells and in an induced model of progeria. She is a postdoctoral research fellow in the MRC Human Genetics Unit at the Institute of Genetics and Molecular Medicine at the University of Edinburgh.

## Notes

**Tamir Chandra** received his PhD from the University of Cambridge and went on to doing a postdoc on chromatin dynamics in cellular senescence at the Babraham and Sanger Institutes. He has a long-standing interest in applying systems biology and genomics approaches to questions in ageing and cancer. Tamir runs a research group at the MRC Human Genetics Unit and is a Chancellor’s Fellow at the University of Edinburgh.

## Notes

**Kristina Kirschner** received her PhD from the University of Edinburgh characterizing one of the first premature ageing mouse models. Her interest in ageing continued throughout her postdoc at the CRUK Cambridge Institute and at the Department of Haematology at the University of Cambridge, where she worked on the functional genomics of p53 in senescence and on haematopoiesis in ageing using single-cell approaches. She moved to the Institute of Cancer Sciences in Glasgow, continuing her work in the fields of haematology, single-cell approaches and ageing.
